# Molecular Diffusion and Self-Assembly: Quantifying the Influence of Substrate hcp and fcc Atomic Stacking

**DOI:** 10.1021/acs.nanolett.2c02895

**Published:** 2022-10-05

**Authors:** Matthew Edmondson, Alex Saywell

**Affiliations:** School of Physics and Astronomy, The University of Nottingham, NottinghamNG7 2RD, United Kingdom

**Keywords:** Diffusion, self-assembly, scanning probe microscopy, Au(111) herringbone reconstruction, Arrhenius analysis

## Abstract

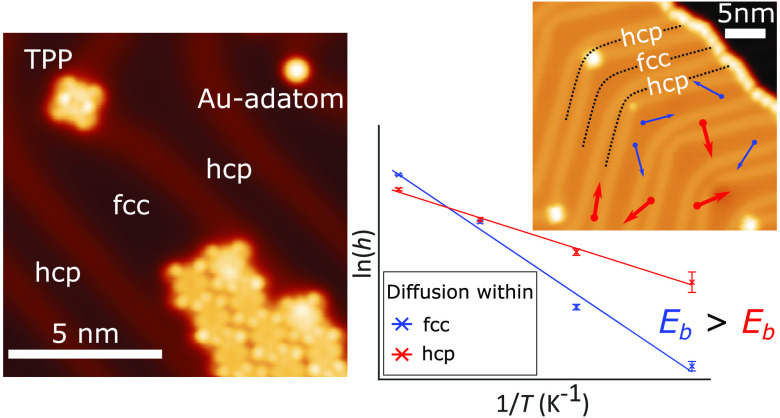

Molecular diffusion is a fundamental process underpinning surface-confined molecular self-assembly and synthesis. Substrate topography influences molecular assembly, alignment, and reactions with the relationship between topography and diffusion linked to the thermodynamic evolution of such processes. Here, we observe preferential adsorption sites for tetraphenylporphyrin (2H-TPP) on Au(111) and interpret nucleation and growth of molecular islands at these sites in terms of spatial variation in diffusion barrier driven by local atomic arrangements of the Au(111) surface (the 22× √3 “herringbone” reconstruction). Variable-temperature scanning tunnelling microscopy facilitates characterization of molecular diffusion, and Arrhenius analysis allows quantitative characterization of diffusion barriers within fcc and hcp regions of the surface reconstruction (where the in-plane arrangement of the surface atoms is identical but the vertical stacking differs). The higher barrier for diffusion within fcc locations underpins the ubiquitous observation of preferential island growth within fcc regions, demonstrating the relationship between substrate-structure, diffusion, and molecular self-assembly.

Self-assembly and on-surface reactivity of molecular species are fundamental to the fabrication of devices which incorporate the functionality of molecular components.^1^ Materials with specific catalytic, electronic, optical, and/or magnetic properties can, in principle, be realized by an appropriate choice of molecule–substrate systems.^2^ The preparation of such materials, however, requires an understanding of the processes which give rise to ordered molecular arrays and, in the case of covalent organic frameworks (COFs), the mechanisms underlying the observed on-surface chemistry.^3^ Scanning probe microscopy (SPM) approaches allow on-surface processes to be studied on the single-molecule and single atom level,^4^ and studies utilizing non-contact atomic force microscopy (nc-AFM) have provided chemical-bond level resolution.^5^ In addition, SPM techniques combined with photoemission spectroscopies can provide detailed structural and chemical characterization of on-surface processes.^6−8^

The temporal evolution of on-surface diffusion has been studied via a variety of techniques^9^ with Arrhenius-based analysis of sequential SPM “images” providing the temperature dependent rates of on-surface processes from which activation barriers and thermodynamic quantities may be obtained under UHV^10^ and liquid^11^ conditions. Examples of such quantities include the energy barriers for molecular diffusion^12,13^ and rotation.^14^

Substrate topography plays a crucial role in on-surface processes, with SPM techniques facilitating characterization of both molecular species and surface features. Specifically, SPM studies have demonstrated that step-edges and surface reconstructions affect molecular diffusion,^10^ orientation,^15,16^ and reactivity.^10,17,18^ Molecular templates have also been employed to influence on-surface diffusion and reactivity.^17,19^ Understanding the interplay between topography and surface-confined processes is a key prerequisite to influence and control molecular structure formation and on-surface chemistry.

The Au(111) surface is frequently employed as a support for molecular self-assembly and on-surface reactions. The shorter surface Au–Au bond length compared to that within the bulk, and the reduction in surface free energy provided by the AB/ABC packing of atomic layers parallel to the surface plane, results in atomic reconstruction where surface atoms buckle to form the characteristic 22× √3 herringbone structure.^20,21^ Nucleation of atomic^22,23^ and molecular^24−26^ species within the face centered cubic (fcc) and hexagonally close packed (hcp) regions of the reconstruction have previously been observed, and the surface itself can initiate reactions. Interestingly, differences in the reactivity and surface interaction energies at the fcc and hcp sites, as calculated by DFT,^27^ may lead to preferred nucleation/reaction sites^28^ (similar to the observation that Cu adatoms, and larger clusters, prefer fcc over hcp sites on Cu(111)^29^). The reaction products of on-surface synthesis have also been reported to be ordered by the Au reconstruction.^30−32^ Here, we investigate the preferential growth of molecular islands within fcc regions by characterizing the rate of diffusion, and island morphology, of free base tetraphenylporphyrins (TPP) on the Au(111) surface over a range of temperatures. By employing Arrhenius analysis we quantify the diffusion barrier for TPP within the fcc and hcp regions of the surface and describe a rationale for the observed preferential island growth within the fcc regions.

Submonolayer coverages of TPP were prepared on a Au(111) single crystal held under ultrahigh vacuum (UHV, base pressure 3 × 10^–10^ mbar), at room temperature, via sublimation and characterized with scanning tunnelling microscopy (STM) at 4.7 K; see SI for experimental details. At very low coverages (∼0.04 ML), step-edge sites and point dislocations on type *x* solitons^33^ [the end point of atomic rows which are incorporated into the surface layer as part of the reconstruction; leading to Burgers circuits with nonzero Burgers vectors^34^] are decorated with TPP (see Figure 1a and SI) with no close-packed structures observed. Increasing the coverage to ∼0.2 ML results in the formation of close-packed islands (as previously reported^8,35,36^), the presence of individual TPP at nearly all point dislocation elbow sites of the herringbone reconstruction and, infrequently, within the fcc or hcp regions of the herringbone reconstruction (see Figure 1b).

**Figure 1:**
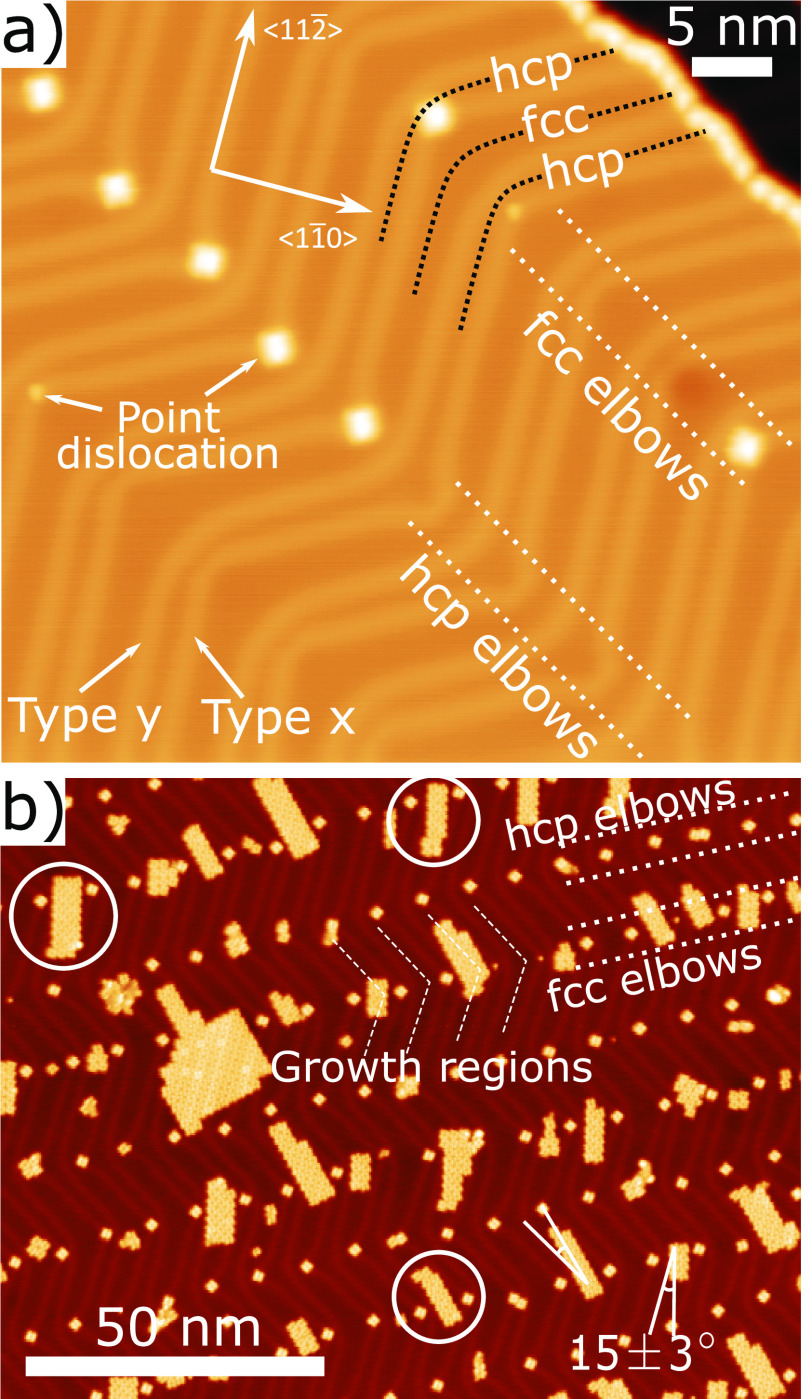
(a) STM image of a ∼ 0.04 ML preparation of TPP on Au(111) (*V*_bias_ = −1.8 V, *I*_set_ = 50 pA). The herringbone reconstruction is clearly visible; the bright lines (solitons) can be divided into type *x* (with point dislocations at elbows where TPP adsorb) or type *y* (with no dislocations). Solitons separate fcc and hcp regions. We define fcc elbows as elbow regions with more fcc than hcp area and vice versa. (b) STM image of ∼0.2 ML preparation of TPP (*V*_bias_ = 2.0 V, *I*_set_ = 20 pA). Small close-packed islands form almost exclusively within fcc sites of the fcc elbows (“growth regions”). The ends of these islands can be seen to laterally shift to stay within the fcc regions of the surface (circled). Larger islands appear to grow into the hcp regions of the surface. The islands are orientated approximately ±15^*o*^ from the soliton direction.

The small close-packed islands of TPP display anisotropic growth, constrained within the fcc regions of the reconstruction. We identify two distinct types of elbow regions, which we label as fcc elbows (*B*_fcc_) and hcp elbows (*B*_hcp_), see Figure 1a, where a local distortion (“pinching” and/or “bulging”) of the solitons gives rise to an increase in the area covered by either the fcc or hcp regions, respectively. Our STM data indicates the *B*_fcc_ has a “growth region” (see Figure 1b.) that promotes the formation of TPP islands, something that is not typically seen in the *B*_hcp_ regions. The ordering of molecular species within the herringbone reconstruction has been observed frequently,^24−26,30−32,37^ but to the best of our knowledge the mechanisms driving the formation of anisotropic growth have not been characterized with respect to the energy barriers for diffusion.

It is clear that islands form preferentially in fcc regions, with the long axis of the islands orientated at 15 ± 3^o^ to the bright line of the herringbone reconstruction (⟨1,1,2̅⟩ directions). These rectangular islands typically contain 2–3 TPP species across the short axis; limited by the area within the fcc regions (at *B*_fcc_). TPP molecules can be observed to be laterally displaced to maintain island growth within the fcc area (see Figure 1b [circled] and SI). Indeed, when the Au reconstruction is locally disrupted, such that the area of the fcc region is increased, TPP islands fill this fcc region and exhibit an island aspect ratio closer to 1 (see SI) while still avoiding hcp regions. The internal structure of the islands are similar to that previously reported, with measured STM overlayer cell dimensions of 1.46 ± 0.05 nm × 1.49 ± 0.10 nm, 90 ± 3° (see SI for details).^8^ Island growth is observed to extend into hcp regions, attributed to instances where the local availability of TPP molecules results in the saturation of the growth region area. We postulate that angularly aligned islands in neighboring fcc regions may stabilize growth in the interstitial hcp regions. In all cases, it is clear that the structure of the herringbone reconstruction drives the alignment and distribution of the molecular islands.

The dynamics of diffusion and island growth for a submonolayer coverage (∼0.16 ML) of TPP on the Au(111) surface were explored via a series of STM measurements with the sample held at temperatures in the range 4.7–285 K (see SI for details). Figure 2 illustrates the temperature of the sample as a function of time, with the plateau regions indicating where the temperature was allowed to stabilize for image acquisition. For sample temperatures in the range 285–205 K (see insets in Figure 2), molecular diffusion is on a time scale faster than that of image acquisition and only surface features such as the herringbone reconstruction and step-edges are resolved; discontinuities and “noise” in the images are indicative of molecular diffusion. At *T*_sample_ = 165 K, TPP islands are visible (Figure 2) which exhibit a close-packed structure with a square lattice. The diffuse appearance at the edges of the island suggests that continuous diffusion is occurring to/from the island. Features are also observed at the point dislocation sites of the herringbone reconstruction, where individual TPP species are absorbed. Reducing the temperature to 125 K confirms the presence of TPP at point dislocation sites, and smaller islands (relative to those present at 165 K) are observed which are in line with the expected dependence of critical island size on temperature.^38,39^ At *T*_sample_ = 4.7 K stable islands are observed (as detailed above) with a significant number of isolated TPP species adsorbed within the fcc (∼80–90% of individual TPP) and hcp regions. The slower cooling rate for a sample imaged at several temperatures in the range 285–4.7 K, as compared to one cooled directly from room temperature to 4.7 K, may underlie the observed prevalence for isolated, kinetically trapped, TPP species (see SI for cooling rate details).

**Figure 2:**
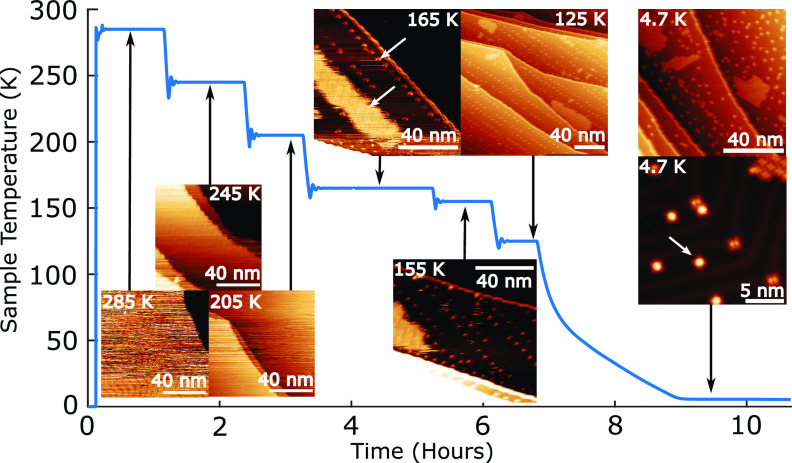
Sample temperature, with corresponding STM data, as a function of time (sample initially at room temperature, with discontinuity indicating sensor being connected). STM images show freely diffusing TPP at 285–205 K; mobile islands at 165–155 K with TPP adsorbed at point dislocation sites on herringbone; stable islands and TPP at point dislocations at 125 K; and coexisting stable islands and individual TPP within both fcc and hcp regions at 4.7 K. All images (except close-up image) scan settings were *V*_bias_ = 0.5 V, *I*_set_ = 10 pA, close-up 4.7 K (bottom right) image settings were *V*_bias_ = 0.4 V, *I*_set_ = 630 pA. See SI for larger versions of these images.

To characterize the energy barrier for diffusion for individual TPP species within the fcc and hcp regions, a temperature dependent rate of diffusion events was calculated. At *T*_sample_ = 20 K diffusion events were infrequently observed, while at 30 K the diffusion rate is faster than the image acquisition time. STM images were therefore obtained for *T*_sample_ = 20, 22, 24 and 26 K, and in each case X,Y and Z thermal drift was minimized before recording a sequence of images (numbering ∼180–210) over ∼20 h (see SI for experimental details and image processing) from which the temperature dependent rate could be obtained. Figure 3a shows example sequential STM images with the position of diffusing molecules highlighted. The STM images in Figure 3 reveal two distinct molecular contrasts (bright/dark) which are assigned to TPP within fcc/hcp regions (bright) and TPP pinned at the point dislocation sites on the herringbone reconstruction (dark). Over the temperature range investigated diffusion of the pinned TPP was not observed, likely due to an enhanced adsorption energy at these sites. It should be noted that this bright/dark contrast is distinct from that previously reported and assigned to the presence of Au-adatoms below TPP,^35^ which we also observe under alternative imaging conditions within close-packed islands (see Figures 5 and S6) but not for the isolated TPP which is the subject of our diffusion measurements.

**Figure 3:**
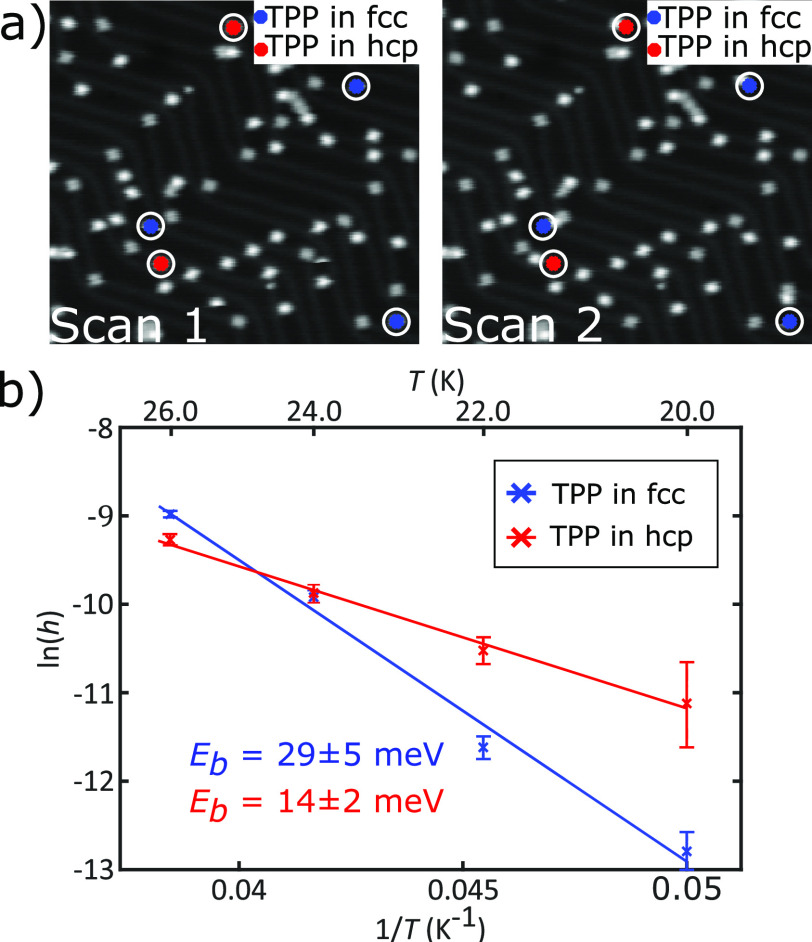
Diffusion Analysis of TPP on Au(111). (a) Cropped sequential planarised STM images showing the detected movements of five TPP molecules in fcc (three moved) and hcp (two moved) sites. Image streaks/part molecules are filtered from the data to minimize false-positive/false-negative counts. All images *V*_bias_ = 0.5 V, *I*_set_ = 5 pA. (b) An Arrhenius plot of ln(*h*) as a function of 1/*T* for diffusion on both the fcc and hcp regions of the Au(111) herringbone reconstruction.

To convert diffusion events per image to a molecular hopping rate, *h*, the total number of TPP features in an image, *N*, and the number of TPP features observed to be at the same location in the subsequent image (i.e., no diffusion occurs), *n*_0_, were counted. Using the ratio of these observations we can obtain *h*
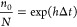
1where *Δt* is the time interval observed; the scan acquisition time (∼380 s). The mean value of *h*, calculated at each temperature, is equated to the rate, *k*, in the Arrhenius equation
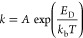
2where *A* is the exponential prefactor, commonly called the attempt frequency, and *T* is the substrate temperature. The experimentally determined energy barrier for diffusion, *E*_*D*_, can be obtained by plotting ln(*h*) as a function of 1/*T*(Figure 3b). By considering hopping rates for TPP within fcc and hcp regions of the surface, the energy barrier to diffusion for molecules in each specific region of the surface can be obtained. There is a significant difference between the diffusion barrier of TPP at fcc sites, *E*_D_ = 29 ± 5 meV, and hcp sites, *E*_D_ = 14 ± 2 meV (the diffusion barriers reported for porphyrin species on Cu(111) are significantly higher, e.g., 0.96 eV^40^ and 0.71 eV,^13^ with the difference with respect to the values reported here attributed to the increased reactivity of the Cu substrate as compared to Au). We propose that the difference in diffusion barrier (between fcc and hcp regions) underlies the preferential formation of molecular islands within the fcc growth regions. As TPP molecules may diffuse between fcc and hcp regions, and as there is a higher barrier to diffusion for TPP at fcc sites (corresponding to a lower rate of diffusion), one would expect an increased residence time within the fcc regions resulting in an increased likelihood of island nucleation (similar to the lower “diffusion potential” reported for benzene within fcc regions of Au(111)^41^). Hence, we propose that the ordered self-assembly of molecular structures on Au(111) is driven by the local difference in diffusion barrier at the fcc and hcp regions of the surface.

Arrhenius analysis also yields values of the prefactor, *A*, which for TPP within the fcc and hcp regions is calculated to be 6 × 10^1±1^ Hz and 4 × 10^–2±1^ Hz, respectively. For simple (monatomic/diatomic) systems a value on the order of 10^13^ Hz is typically expected, and values of the order 10^9^ to 10^13^ have been obtained for porphyrin species on Cu(111).^13,40^ Interestingly, anomalously low values of *A* have been reported where diffusion barriers are below 100 meV,^42^ as is the case here, including 2 × 10^3±1^ Hz for Al on Au(111).^43^ For smaller molecules (e.g., CO) quantum tunnelling processes have been suggested as an explanation for low frequency values,^44^ but are unlikely to contribute here due to the comparatively high molecular weight of TPP. The values reported here should be considered in light of (i) the nontrivial assignment of an expected prefactor. As recently discussed,^45^ viewing *A* as simple “attempt frequency” to the transition state is potentially an oversimplification for large/flexible molecules, and the change in partition function, with associated entropic considerations, is likely to be relevant. (ii) The diffusion lengths recorded here are greater than the lattice spacing of the substrate, suggesting that the transition state accessed here may not be directly comparable to systems where smaller displacements are observed.

It should be noted that while STM offers single-molecule resolution and facilitates the measurment of individual diffusion events, there is potential for the STM tip to interact with molecular species and enhance/suppress diffusion. When taking STM measurements, the tip is raster scanned over a small area of the surface, and the tip will therefore pass over single molecules multiple times during a image. It is conceivable that not only direct tip-molecule interaction moves the molecule,^46^ but also that the applied bias can play a role in inducing motion.^47^ Previous STM Arrhenius studies have quoted that tunnel resistances between 1 and 10 GΩ are sufficient to prevent tip interaction,^12,13^ we employ a resistance of 100 GΩ (500 mV and 5 pA) to reduce the likelihood of tip-induced processes; providing a significant tunnelling resistance while limiting the magnitude of the electric field present in the tip–sample junction. We do not exclude the possibility of tip-induced processes, but the simultaneous measurement of diffusion barriers for fcc and hcp regions allows a direct comparison under the same tip conditions.

In conclusion, the high spatial resolution of our variable-temperature STM measurements allows the diffusion rates (and associated energy barriers) for site-specific diffusion over the Au(111) substrate to be characterized. Preferential formation of atomic and molecular islands within the fcc regions of the Au(111) herringbone reconstruction is ubiquitous, and the observed difference in diffusion energy barrier between TPP molecules within fcc and hcp may offer an explanation for the underlying mechanism. Consideration of molecular diffusion barriers, driven by local substrate atomic order, offers a route to control the spatial distribution and orientation of on-surface self-assembled structures and reactions.

## Supporting Information Available

Details of experimental methods, additional STM data, details of Arrhenius analysis (PDF)
Video of STM data for TPP diffusion at 20 K (AVI)
Video of STM data for TPP diffusion at 22 K (AVI)
Video of STM data for TPP diffusion at 24 K (AVI)
Video of STM data for TPP diffusion at 26 K (AVI)

## Supplementary Material

nl2c02895_si_001.pdf

nl2c02895_si_002.avi

nl2c02895_si_003.avi

nl2c02895_si_004.avi

nl2c02895_si_005.avi
