# Supplementary material for: Molecular Diffusion and Self-Assembly: Quantifying the Influence of Substrate hcp and fcc Atomic Stacking
Source: Nano Lett. 2022 Oct 5;22(20):8210–5. doi: 10.1021/acs.nanolett.2c02895 (PMC9614974; doi:10.1021/acs.nanolett.2c02895)
Supplement: Supplementary file 1 — nl2c02895_si_001.pdf [file nl2c02895_si_001.pdf]

Molecular diffusion and self-assembly: Quantifying the influence of substrate *hcp* and *fcc* atomic stacking

Matthew Edmondson and *Alex Saywell**

[1]School of Physics & Astronomy, The University of Nottingham, Nottingham, NG7 2RD, UK.

*Corresponding author A.S (Alex.Saywell@nottingham.ac.uk)

Supporting Information

Contents

Experimental Methods S2

Procedures for determining diffusion rate S5

TPP adsorption S7

Controlled cooling of TPP on Au(111) S12

Experimental Methods

STM

Scanning tunnelling microscopy (STM) experiments were performed using a Scienta Omicron POLAR low temperature STM system operating under ultra-high vacuum (UHV) conditions with a base pressure of better than $3x10^{-10}$ mbar. The STM was cooled to liquid helium temperatures, with a sample temperature of 4.7 K. All STM measurements were performed in constant current mode using electrochemically etched tungsten tips that may be coated in gold during tip optimisation by controlled indentation into the Au(111) single crystal substrate.

Sample Preparation

The Au(111) single crystal surface (Surface Preparation Laboratory) was prepared by cycles of Ar ion sputtering for 30 minutes at 1.0 keV, followed by annealing at 770 K for 30 minutes. Annealing sample temperatures were estimated by thermocouple measurements close to the PBN heater stage.

Tetraphenylporphyrin (TPP) was purchased from Merck with a quoted purity of 99.8%. Molecules were first thermally purified by degassing at 200°C for several hours prior to deposition. The molecules were deposited (using a Kentax UHV Evaporator heated to 225°C) onto the Au(111) surface that was held at room temperature. Coverage of TPP was altered by increasing/decreasing deposition time following surface cleaning.

VT-STM Measurements

In the POLAR STM, the sample was heated *via* an integrated sample plate heater. Sample temperature was monitored via a LakeShore temperature controller (using a Si temperature diode on the plate heater). The controller also acted as a PID controller to set and maintain the sample temperature at a

steady state to within 0.01 K. Actual sample temperature may differ from the set temperature, estimated by Scienta Omicron to be <1K. The PID gains settings were optimised to minimise over/undershoot whilst reaching the set temperature quickly.

For the Arrhenius diffusion measurements, scanning was commenced a minimum of one hour after the sample had reached the set temperature to minimise X/Y/Z thermal drift. Furthermore, to minimise the effect of 'piezo creep', the first few scans of a new area were not used in the analysis of molecular diffusion. For the STM images recorded during counter heating/cooling of the sample, the surface was left to thermalise for at least 10 minutes after the temperature set-point was reached before image acquisition commenced.

As the sample is counter heated (with the bath LHe cryostat cooling the sample), the surrounding area of the STM head is also heated until thermal equilibrium is reached. This has the effect of reducing the effective cooling power on the sample, therefore, the sample cools to 4.7K more slowly, taking ~2.1 hours (if counter heated first at 125K), than if the sample was just cooled immediately from 280K to 4.7K; taking ~1.1 hours. The difference in cooling rates is shown in Fig. S1.

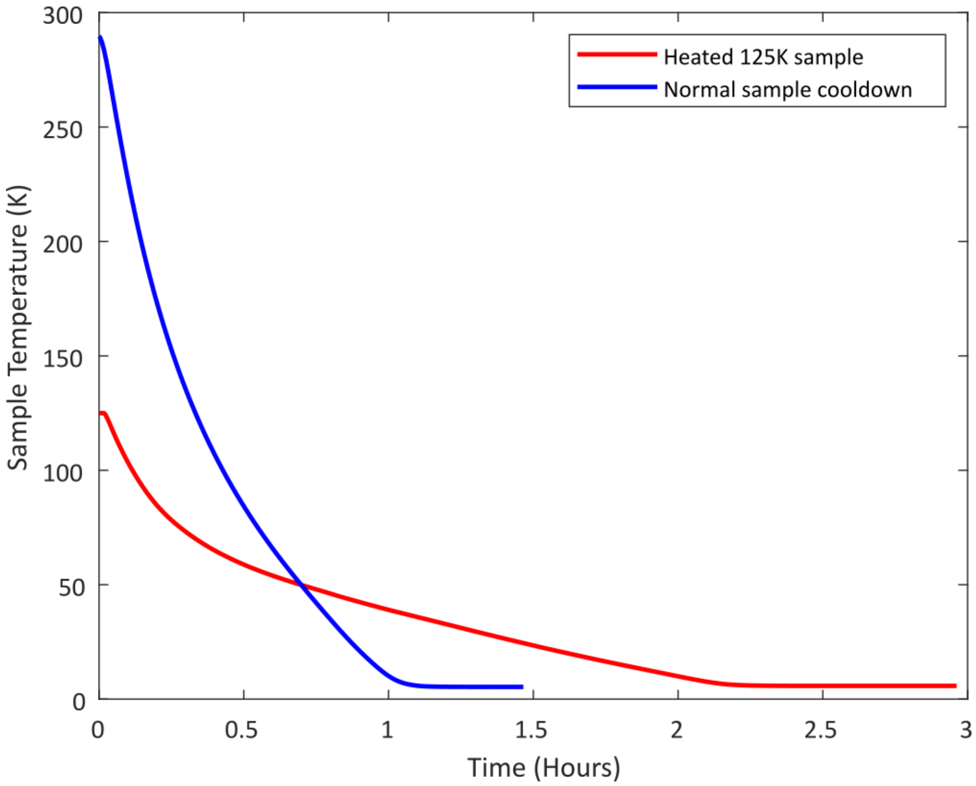

Figure S1: Temperature change as a function of time for an Au sample cooling to 4K for; (i) a sample that has been counter heated to maintain a sample temperature of 125K (red), and (ii) the same sample that was at room temperature prior to being cooled to 4K immediately upon insertion into the STM (blue).

Procedures for determining diffusion rate

Details of image processing

Despite efforts to reduce drift during scanning, minimal drift between consecutive images was observed. This was removed within the sequence of images analysed by the following process. A three-point plane subtraction was applied to each image, followed by thresholding such that the Au(111) herringbone reconstruction was not visible in the image. A stationary object in an image is used as a reference point to align all subsequent images. Due to a combination of the piezoelectric response of the SPM scan tube, and thermal effects, the lateral resolution available to determine whether a diffusion event has occurred is limited to 0.6 nm. It is therefore likely that diffusion events on the length scale of single atomic sites are not fully captured and that the diffusion events recorded are 'long' jumps over several atomic sites.

For STM data recorded at each temperature the image stabilisation process was performed. A 'video' of the sequential STM images acquired for each sample temperature (20 K [173 images], 22 K [191 images], 24 K [207 images] and 26 K [200 images]) can be found in the supplemental data.

Table S1: Details of the 'videos' (sequential STM images) used to analyse molecular diffusion. The number of TPP molecules in a single STM image at a given sample temperature is provided, with the total number of molecules analysed for a given temperature being approximately equal to, No. of images – 1 × No. of TPP (single image).

**Sample Temperature (K)**	**No. of images in 'video'**	**No. of TPP identified (single image)**	**No. of TPP identified in *fcc* locations (single image)**	**No. of TPP identified in *hcp* locations (single image)**
20	173	111	107	4
22	191	132	112	20
24	207	96	83	13
26	200	122	104	18

Counting motion

As the herringbone structure had been previously removed from the data using image height thresholding, the centre position and size of the molecular features was able to be acquired. By comparing consecutive images, it is possible to determine if the molecule had moved by ascertaining

whether the position of the molecule had changed. Due to the drift/piezo derived limitations on lateral resolution a cut-off of 1.0nm was defined as the minimum movement distance required to count as a diffusion event. To reduce false positive counts, an automated procedure was employed to checked if a molecule was in the same position in the image after a suspected diffusion event (i.e. if a molecule identified in frame *n* was judged to have diffused to a new location in frame *n+1*, the position of this molecule in frame *n+2* was compared to that in *n* to ensure genuine motion had been detected). Repeated diffusion events between two locations were deemed to be unlikely.

Estimation of errors

For each pair of images within the 'video' the diffusion rate was counted using equation 1 (see main manuscript) and an average of these values was used to determine the average diffusion rate for TPP at a given temperature. The standard deviation for this distribution is used as the error and propagating the error results in the y-direction error bars shown in Figure 3. The estimated error in the temperature (based upon the fluctuation in temperature) is ± 0.01 K and as such error bars in the x-direction have a similar dimension to that of the data point symbols (hence error bars are not plotted in the y-direction).

TPP adsorption

Low sub-monolayer coverages (~0.04ML)

At this coverage, no close-packed island structures were found. TPP was observed to adsorb primarily along the step-edges of the Au(111) terraces and on the type 'x' soliton point-dislocation sites of the herringbone reconstruction elbows (see Fig S2).

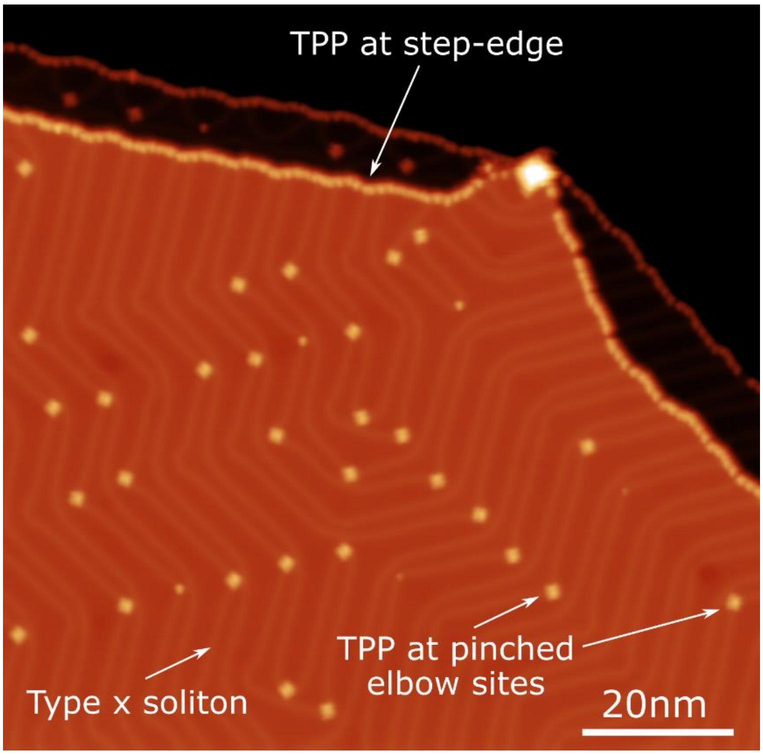

Figure S2: Low sub-monolayer coverage of TPP on Au(111). TPP is found adsorbed on point-dislocation elbow sites (x soliton) and along step-edge features. At this coverage, not all elbow sites have TPP adsorbed. The TPP on the step-edge appear to sit partially on both terraces. Image settings: V_{bias} = - 1.8 V, I_{set} = 50 pA and T_{sample} = 4.7 K.

Sub-monolayer coverage (~0.2 ML)

At higher coverages, close-packed islands of TPP form within *fcc* elbow 'growth regions'. The morphology of the close-packed islands are influenced by the Au herringbone reconstruction. A large (high resolution) STM image is shown in Figure S3.

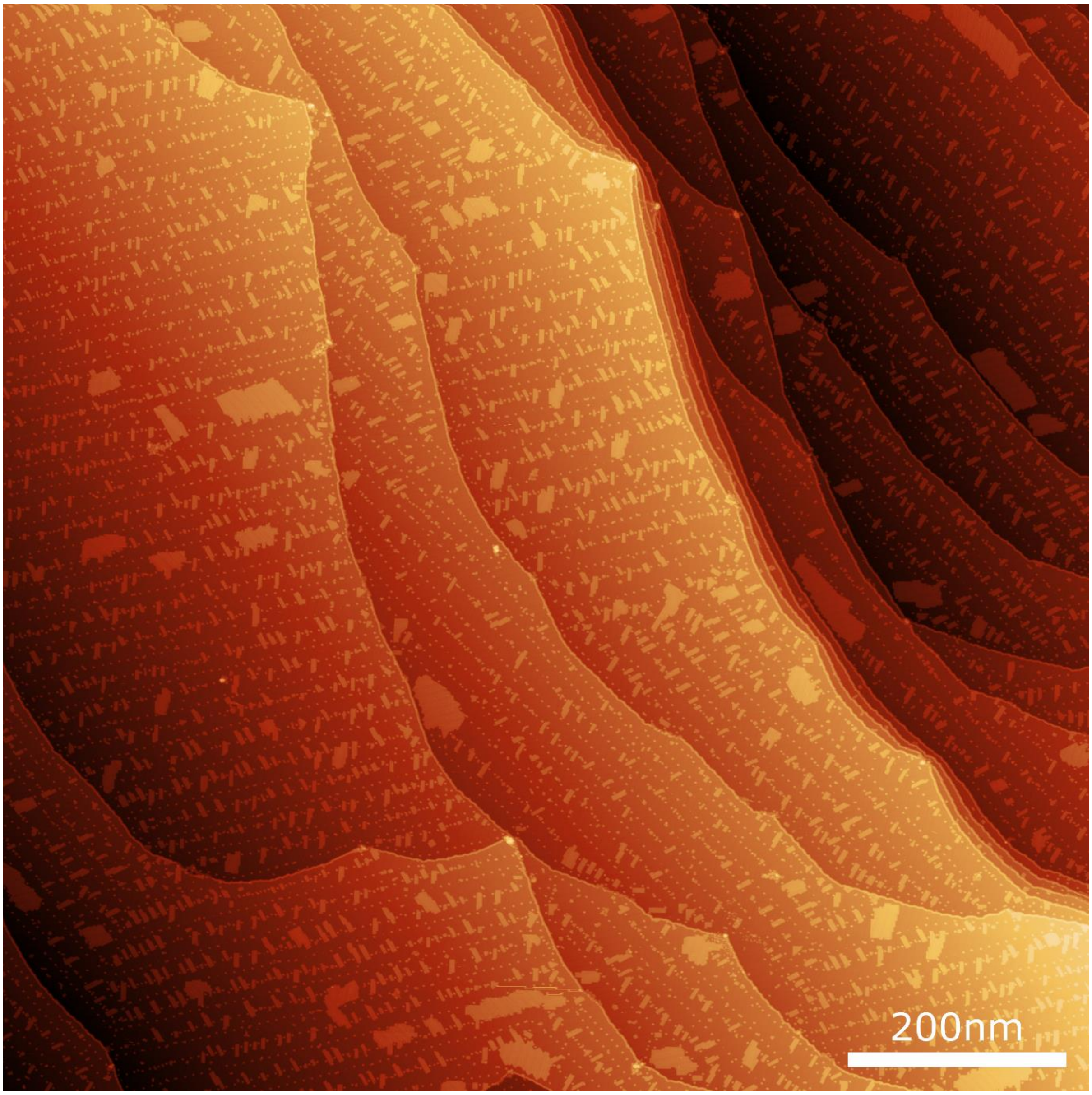

Figure S3: Large scale image of island growth of TPP on Au(111). Small rectangular islands preferentially form within *fcc* elbow growth regions. Larger islands in general appear to be confined to between the elbow regions, on the 22x$\sqrt{3}$ reconstruction. Image settings: V_{bias} = 2.0 V, I_{set} = 20 pA and T_{sample} = 4.7 K.

Similar to the low sub-monolayer coverage (~0.04 ML), TPP occupies the edge of the Au(111) terrace and almost all point defects of the x solitons. Small, rectangular (2-3 molecules on the short axis) TPP islands formed on the *fcc* elbow regions. TPP islands are very seldomly found in the hcp elbow regions (see alternating rows of *fcc* elbow regions with island growth and hcp elbow regions with no islands [see Fig. S4]). These small rectangular islands are aligned at ± 15±3° from the $< 1,1,\bar{2} >$ direction

(bright herringbone lines) and appear to either not enter the *hcp* region by their limited size or, the ends of the islands are offset (compared to the rest of the island) to avoid the *hcp* region (see Fig. S4 – white arrows).

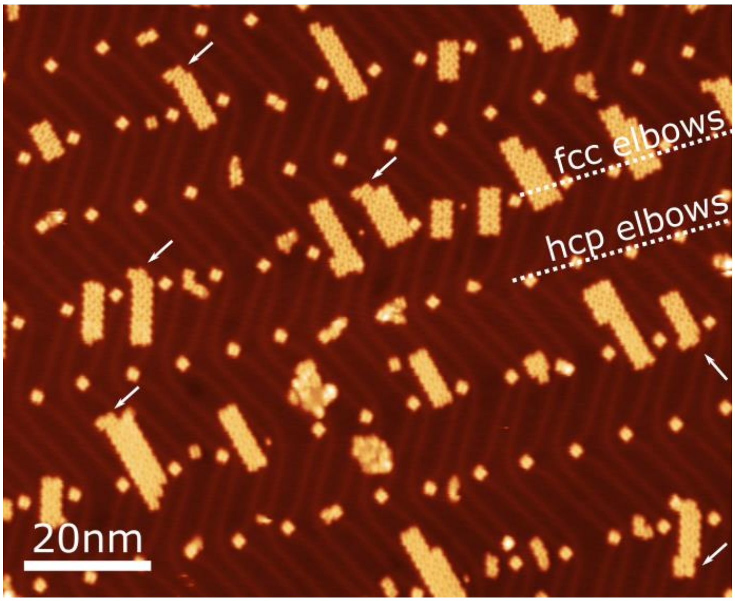

Figure S4: Alternating rows of elbows have island growth (preferential island growth in *fcc* elbows, infrequent island growth in *hcp* elbows). Some smaller rectangular islands are offset at the end (arrows point to TPP molecules that have shifted) to avoid entering the hcp region. Islands are orientated along the *fcc* region and are limited in size as the *hcp* sites of the herringbone constrain the possible width/size of island. Image settings: V_{bias} = 2.0 V, I_{set} = 20 pA and T_{sample} = 4.7 K (cropped image from Fig. S3).

Larger islands are not constrained to only *fcc* sites/ growth regions alone, but are found in both *fcc* and *hcp* sites (as expected from monolayer coverages of molecules on Au(111) (see Fig. S5a). Interestingly, these islands, in general, do not grow beyond the *hcp* elbow sites (see Fig. S4). There is one example where the island has grown beyond the *hcp* elbow (Fig. S5b), this may require rare, but possible, island formation at the *hcp* elbow to enable this growth.

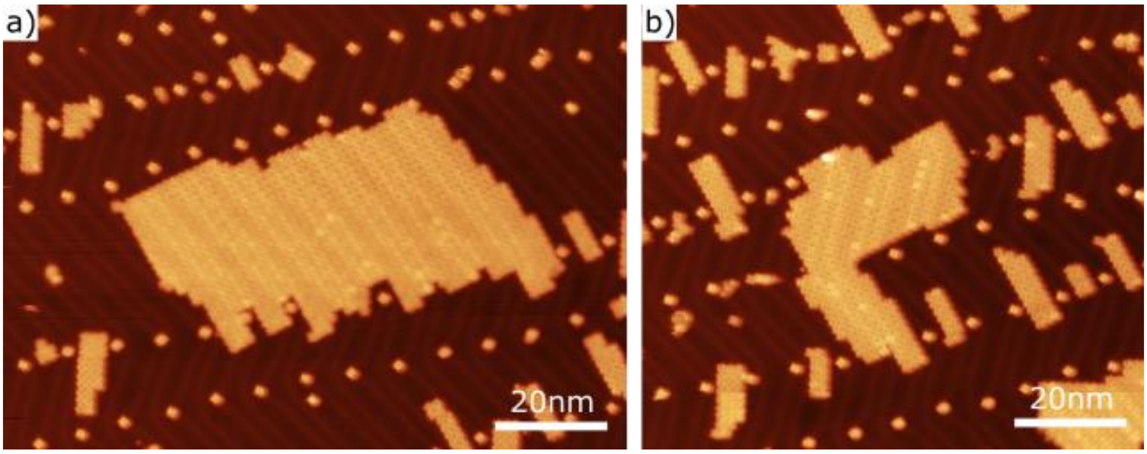

Figure S5: Large islands form between elbow sites on the 23 x $\sqrt{3}$ section regions. (a) Growth beyond the elbow regions are possible on the *fcc* regions. (b) Rarely, growth beyond the *hcp* region is seen. Image settings: V_{bias} = 2.0 V, I_{set} = 20 pA and T_{sample} = 4.7 K (cropped image from Fig. S3).

Local changes to the herringbone reconstruction can coincide with larger *fcc* regions on the surface (see Fig. S6). The TPP islands in these regions are not limited to 2-3 molecule wide islands; showing that the islands sizes are initially limited by the *hcp* region of the surface.

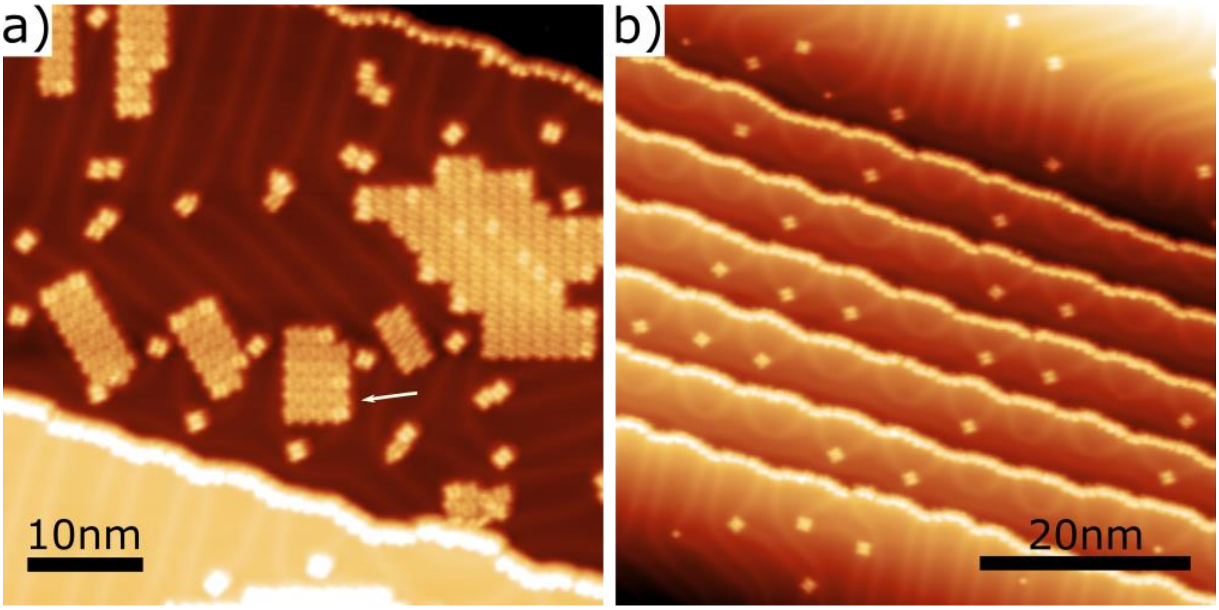

Figure S6: a) Small island growth is shown within the *fcc* region of the surface. When the herringbone reconstruction differs from the common type 'x' and type 'y' soliton pattern (compare to Fig. S2 and Fig. S6b), the *fcc* region shown by the arrow has increased in size. This has allowed wider than 2-3 TPP small islands to form, filling the total space given by the *fcc* region. b) Shows the variation in the herringbone reconstruction that is driven by step-edge features on a ~0.04ML coverage of TPP/Au(111) sample. Image settings: a) V_{bias} = 2.0 V, I_{set} = 20 pA and T_{sample} = 4.7 K, b) V_{bias} = -1.8 V, I_{set} = 50 pA and T_{sample} = 4.7 K.

TPP Close-packed island structure

Similar to previously published packing arrangements, the dimensions of the lattice for close-packed TPP found in this work are: a = 1.46 ± 0.05 nm, b = 1.49 ± 0.10 nm, ϴ = 90 ± 3°

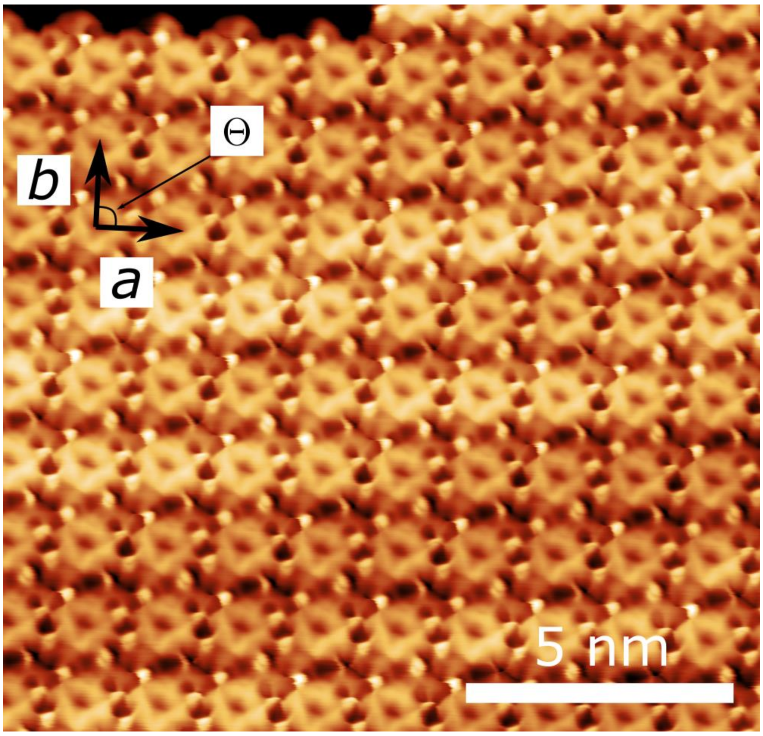

Figure S7: Calibrated image (using Au(111) atomic resolution image) of a close-packed TPP island showing the vectors of the square unit cell. Image settings: a) V_{bias} = 0.45 V, I_{set} = 610 pA and T_{sample} = 4.7 K.

Controlled cooling of TPP on Au(111)

Counter heating sample as cooling to 4 K

The bath LHe STM cryostat was at 4.3 K whilst a sample (at 293 K) was inserted into the STM. Under normal conditions the sample would cool to 4 K within 1 hour (see blue line in Fig. S1). To keep the sample at ~285 K, the sample was counter heated as to maintain a set temperature (see Fig. 2). The images shown in Fig. S8 are larger versions of those shown in the main manuscript.

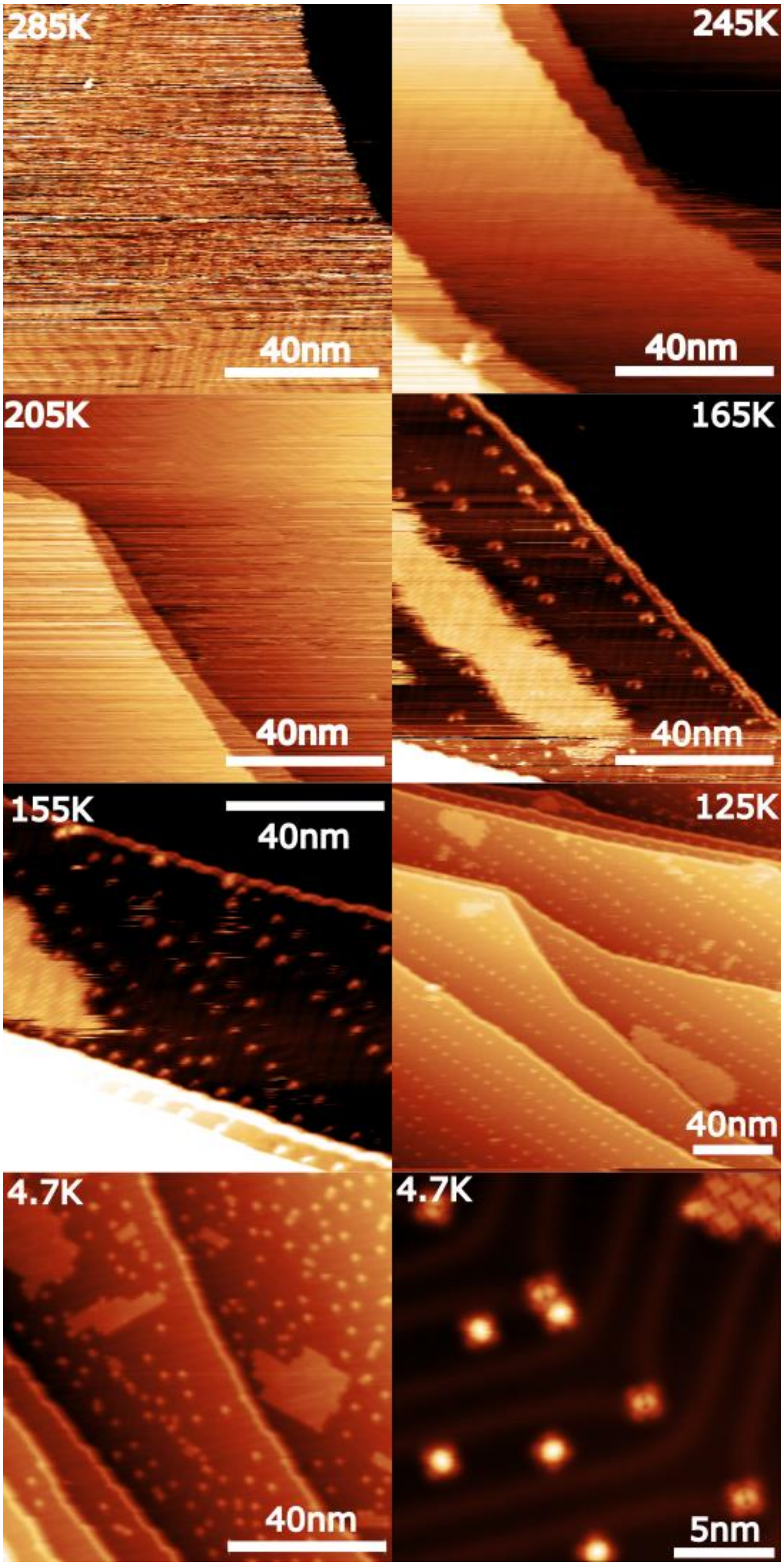

Figure S8: Showing the STM images from each temperature measured. VT-STM image settings: V_{bias} = 0.5 V, I_{set} = 10 pA. Image settings for left 4.7K image: V_{bias} = 0.5 V, I_{set} = 10 pA and right 4.7 K image: V_{bias} = 0.4 V, I_{set} = 630 pA.
